# Negative regulation of mitochondrial transcription by mitochondrial topoisomerase I

**DOI:** 10.1093/nar/gkt768

**Published:** 2013-08-27

**Authors:** Stefan Sobek, Ilaria Dalla Rosa, Yves Pommier, Beatrice Bornholz, Faiza Kalfalah, Hongliang Zhang, Rudolf J. Wiesner, Jürgen-Christoph von Kleist-Retzow, Frank Hillebrand, Heiner Schaal, Christian Mielke, Morten O. Christensen, Fritz Boege

**Affiliations:** ^1^Institute of Clinical Chemistry and Laboratory Diagnostics, Heinrich-Heine-University, Med. Faculty, D-40225 Düsseldorf, Germany, ^2^Laboratory of Molecular Pharmacology, Center for Cancer Research, National Cancer Institute, National Institutes of Health, Bethesda, MD 20892, USA, ^3^Center for Physiology and Pathophysiology, Institute of Vegetative Physiology, University of Köln, D-50931 Köln, Germany, ^4^Center for Molecular Medicine Cologne, University of Köln, D-50931 Köln, Germany, ^5^Cologne Excellence Cluster on Cellular Stress Responses in Aging-associated Diseases, University of Köln, D-50931 Köln, Germany, ^6^Department of Pediatrics, Med. Faculty, University of Köln, D-50931 Köln, Germany and ^7^Center for Microbiology and Virology, Institute of Virology, Heinrich-Heine-University, Med. Faculty, D-40225 Düsseldorf, Germany

## Abstract

Mitochondrial topoisomerase I is a genetically distinct mitochondria-dedicated enzyme with a crucial but so far unknown role in the homeostasis of mitochondrial DNA metabolism. Here, we present data suggesting a negative regulatory function in mitochondrial transcription or transcript stability. Deficiency or depletion of mitochondrial topoisomerase I increased mitochondrial transcripts, whereas overexpression lowered mitochondrial transcripts, depleted respiratory complexes I, III and IV, decreased cell respiration and raised superoxide levels. Acute depletion of mitochondrial topoisomerase I triggered neither a nuclear mito-biogenic stress response nor compensatory topoisomerase IIβ upregulation, suggesting the concomitant increase in mitochondrial transcripts was due to release of a local inhibitory effect. Mitochondrial topoisomerase I was co-immunoprecipitated with mitochondrial RNA polymerase. It selectively accumulated and rapidly exchanged at a subset of nucleoids distinguished by the presence of newly synthesized RNA and/or mitochondrial RNA polymerase. The inactive Y559F-mutant behaved similarly without affecting mitochondrial transcripts. In conclusion, mitochondrial topoisomerase I dampens mitochondrial transcription and thereby alters respiratory capacity. The mechanism involves selective association of the active enzyme with transcriptionally active nucleoids and a direct interaction with mitochondrial RNA polymerase. The inhibitory role of topoisomerase I in mitochondrial transcription is strikingly different from the stimulatory role of topoisomerase I in nuclear transcription.

## INTRODUCTION

Human mitochondrial DNA (mtDNA) is a closed double-stranded DNA circle (1). Strand separation during transcription and replication creates topological stress that interferes with mtDNA metabolism if not released by topoisomerases (2). Two of the three topoisomerases so far found in mitochondria are produced from common genes encoding nuclear and mitochondrial enzyme variants. Mitochondrial topoisomerase IIIα (TOP3A) is produced by alternative translation initiation from a common transcript (3). Mitochondrial topoisomerase IIβ (TOP2B) is derived by limited proteolysis from the nuclear enzyme variety (4). The exception is mitochondrial topoisomerase I (TOP1MT), which is encoded by a separate nuclear gene (5) conserved in vertebrates (6). *TOP1MT*^−^^/^^−^ mouse embryonic fibroblasts (MEFs) exhibit mitochondrial dysfunction and retrograde activation of a nuclear mito-biogenic stress response (7). The nuclear variety of topoisomerase I (TOP1) is incompatible with mtDNA transcription, whereas TOP1MT does not interact with nuclear chromosomes (8). Thus, TOP1MT apparently plays a specific and relevant role in mtDNA metabolism and cell respiration, but the function it performs in these processes remains unclear.

The mitochondrial genome is organized in nucleoids, structures composed of mtDNA (1) and proteins including TOP1MT (9–11). Mammalian nucleoids have a uniform size ∼100 nm in diameter and mostly contain a single mtDNA copy (12). A cluster of TOP1MT DNA-cleavage has been identified in an mtDNA region downstream of the displacement loop (D-loop) (13) that contains an additional DNA strand (7 S DNA). 7 S DNA is either a prerequisite or a side product of mtDNA replication (14). Depletion of 7 S DNA upon inhibition of TOP1MT (13) suggests an involvement in D-loop maintenance or replication, but an essential role of Top1mt in mtDNA maintenance seems unlikely, as *TOP1MT*^−/−^ mice express mtDNA-encoded proteins (7).

In the vertebrate nucleus, TOP1 activity is an essential cofactor of transcription. TOP1 is constitutively associated with RNA polymerase I (15) and rDNA (16) and promotes rRNA-transcription (17) and activator-dependent mRNA-transcription (18,19). Here, we present data, surprisingly suggesting that TOP1MT activity possibly has the opposite function in mitochondrial transcription, as it decreases mitochondrial transcript abundance.

## MATERIALS AND METHODS

Culture of *TOP1MT*^−/−^ MEFs levels followed published procedures (7). For re-complementation, the cDNA of mouse *TOP1MT* (NM_028404.2) was cloned into the retroviral vector pFB-Neo (Stratagene, La Jolla, California, USA) to generate pFB-*TOP1MT*-Neo, which was co-transfected with packaging plasmids (pVPack-GP and pVPack-VSV-G, Stratagene) in 293T cells to produce *MMLV*-based viral particles for transduction of *TOP1MT*^−/−^ MEFs. Virus particles containing empty vector served as negative control. TOP1MT expression was confirmed by immunoblotting 24 h after transduction. To generate MEFs defective in *TOP1MT* and *TOP2B*, *TOP1MT^+/^^−^* mice were crossed with *TOP2B^+/^^−^* mice (20). Pregnant females were sacrificed on day 12.5 of pregnancy, and the embryos were used to generate MEFs. MEFs were obtained from *TOP2B*^−/−^ mice and *TOP2B^+/+^* littermates as published in (20). Absence of TOP1MT and/or TOP2B in these MEFs was ascertained by immunoblotting.

All experiments involving human cells were carried out with the human fibrosarcoma cell line HT1080 (# DSMZ ACC 315, Braunschweig, Germany). For mtDNA depletion, cells were cultivated for 14 days with ethidium bromide (EtBr, 50 ng/ml) and uridine (50 µg/ml). TOP1MT was knocked down by transfection for 48 h with four small interfering RNAs (siRNAs) targeting mRNA sections coding for constitutive regions of human *TOP1MT* (amino acid residues 218–226, 266–284, 715–733 and 1353–1371). A pool of four siRNAs of similar length that have no known target among human mRNA served as negative control. siRNAs were obtained from Dharmacon Inc. (Lafayette, Colorado, USA) and applied at a final concentration of 5 nM each. Stable overexpression of human TOP1MT or TOP1MT^Y559F^ followed published procedures using bicistronic vectors (8). For visualization in live cells, yellow fluorescent protein (YFP) was appended to the N-terminus, and a mitochondrial targeting sequence (MTS) was N-terminally attached to YFP to compensate for masking of the endogenous MTS of TOP1MT. Mitochondria targeted YFP alone served as control. For each construct (schematized in Supplementary Figure S1), at least five independent cell clones with similar expression levels were established.

Abundance of TOP1MT protein or index proteins of respiratory complexes was determined in isolated mitochondria (8) by immunoblotting, chemo-luminescence and luminometry (LAS 4000, Fuji, Düsseldorf, Germany) using YFP antibodies (clone JL8, Clontech, Heidelberg, Germany) or TOP1MT antibodies (13) or antibodies directed against index subunits of respiratory complexes that are unstable when not assembled (see Figure 3C) (Abcam, Cambridge, UK). Gel loading was equalized according to protein content of the samples and controlled by amido black staining of the blotted membranes. For immunoprecipitation, extracts of isolated mitochondria were incubated (30 min, 4°C) with antibodies (3 µg) against mitochondrial RNA polymerase (POLRMT) (sc-67350, Santa Cruz Biotechnology, Santa Cruz, California) or YFP. Immune complexes were isolated using µMACS Protein A/G microBeads or µMACS GFP-tagged Protein Isolation Kit (Miltenyi Biotec, Bergisch Gladbach, Germany) and probed with antibodies against POLRMT (GTX105137, GeneTex, Irvine, CA, USA), mitochondrial transcription factor A (TFAM) (21), YFP or TOP1MT. DNA relaxation activity was determined in extracts of isolated mitochondria as previously described (8).

Imaging of fluorescence in fixed or live cells and photo-bleaching experiments followed published procedures (16). For immune-staining, cells were fixed (3.7% paraformaldehyde in PBS, 10 min, 37°C), made permeable (0.25% TX-100 in PBS, 10 min, 37°C), incubated with antibodies against TFAM (21), POLRMT (ab32988, Abcam, Cambridge, UK), bromo uridine (clone ZBU30, Invitrogen) or DNA (clone AC-30-10, Progen, Heidelberg, Germany) and counterstained with CY2- or CY3-conjugated secondary antibodies (Dianova, Hamburg, Germany). For visualization of nascent transcripts, 2.5 mM bromo uridine (BrU) was added to the culture medium 1 h before fixation (1). Mitochondria were visualized with MitoTracker Red (Invitrogen).

Focal intra-mitochondrial accumulation of fluorescence was quantified by the difference in fluorescence intensity between a focus and the adjacent background, and by the number of foci along a given stretch of mitochondrial tubule. Foci were defined by a contiguous increase above background of >2-fold within three pixels. These parameters were determined in raw data of cells imaged in middle plane by confocal fluorescence microscopy using ImageJ 1.4.2Q (National Institute of Health, Bethesda, USA). The true diameter of fluorescent foci was evaluated by super-resolution microscopy (12) using a Leica TCS SP 8 Stimulated Emission Depletion (STED) microscope (Leica, Mannheim, Germany) equipped with a 100 × oil STED objective. Images in STED mode were acquired with fluorescence lifetime gating.

Quantification of mtDNA, mRNAs of mitochondrial topoisomerases (TOP1MT, TOP2B, TOP3A), mitochondrial transcripts [mitochondrial encoded cytochrome c oxidase I (COX1), mitochondrial encoded cytochrome c oxidase II (COX2), mitochondrial encoded NADH dehydrogenase 2 (ND2), mitochondrial encoded NADH dehydrogenase 5 (ND5), mitochondrial encoded NADH dehydrogenase 6 (ND6), mitochondrial rRNA (12S)], markers for activation of the mito-biogenic program in the nucleus [TFAM, PPARγ-coactivator 1α encoded by the PPARGC1A gene (PGC1A), nuclear respiratory factor 1(NRF1), the α-subunit of GA binding protein complex (GABPA), mtDNA polymerase gamma (POLG)] and succinate dehydrogenase subunit B (SDHB) followed published procedures (7,8). Analysis of transcript length by northern blotting is described in (22). In all, 1, 2 and 3 µg of total cellular RNA were applied to the analysis of 12S, COX1/2 and ND2/5/6, respectively. Digoxigenin (DIG)-labelled probes were amplified from mtDNA using the same primers as for qRT-PCR (23).

Oxygen consumption studies were performed on freshly harvested cells using a Clark type electrode (Hansatech Instruments, King’s Lynn, England) as described in (24). Lactate accumulation in culture media was determined by accredited diagnostic procedures. Superoxide levels were measured by flow cytometry (25) using MitoSox (Invitrogen, Karlsruhe, Germany).

GraphPad PRISM 4.0a (GraphPad Software Inc., USA) was used to analyse normal data distribution (Shapiro–Wilk test) and calculate significances (two-sided Student’s *t*-test). Differences considered statistically significant are marked by *(*P *< 0.05), **(*P*< 0.01) or ***(*P*< 0.001).

## RESULTS AND DISCUSSION

### Active Top1mt has a direct negative effect on mitochondrial transcript abundance

**Figure 1.**
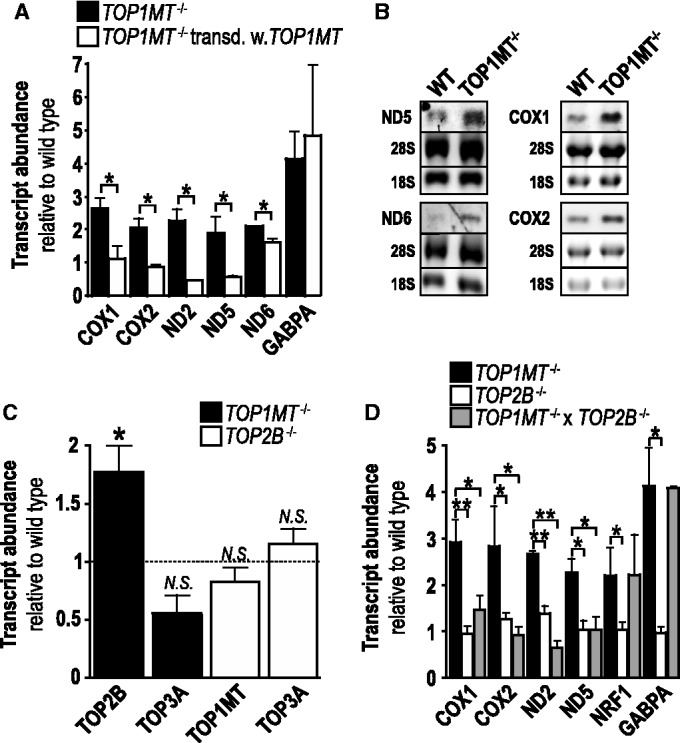
Impact of constitutive TOP1MT and TOP2B deficiency on transcript abundance. (**A**) Quantitative RT-PCR of the indicated transcripts in *TOP1MT*^−/−^ MEFs (black bars) and MEFs re-complemented with TOP1MT by retroviral transduction (white bars). (**B**) Northern blot analysis of the indicated transcripts in wild-type MEFs (left lane) and *TOP1MT*^−/−^ MEFs (right lane). 28 S and 18 S: nuclear rRNA used as loading control. (**C**) Quantitative RT-PCR of TOP1MT, TOP2B and TOP3A mRNA in *TOP1MT*^−/−^ (black columns) and *TOP2B*^−/−^ (white columns) MEFs; dotted line: levels in wild-type MEFs; asterics, N.S.: significance versus wild-type. (**D**) Quantitative RT-PCR of the indicated transcripts in *TOP1MT*^−/−^ MEFs (black bars), *TOP2B*^−/−^ (white bars) and *TOP1MT*^−/−^ × *TOP2B*^−/−^ MEFs (grey bars). All quantitative RT-PCR data are normalized to the values in wild-type MEFs and given as mean ± SEM, *n *= 5. A representative example for northern blots (*n *= 3) is shown in (B). Full-length northern blots are shown in Supplementary Figure S3.

*TOP1MT*^−/−^ MEFs had significantly (2–3-fold) higher levels of mtDNA-encoded transcripts than wild-type MEFs (Figure 1A). The increase was quantitatively similar for transcripts distributed across the entire length of heavy strand transcription (COX1, COX2, ND2, ND5) or expressed under the control of the light strand promoter located in the D-loop (ND6). A trend towards increased mtDNA transcript abundance was also observed in myocardium and skeletal muscle of *TOP1MT*^−/−^ mice (Supplementary Figure S2). It was confirmed by northern blotting that mitochondrial transcripts where more abundant in *TOP1MT*^−/−^ MEFs but had the same electrophoretic mobility as those in normal MEFs (Figure 1B). No aberrant transcripts were ever seen on these blots (Supplementary Figure S3), excluding that TOP1MT deficiency leads to premature or defective termination of transcription. Levels of mtDNA transcripts were normalized in *TOP1MT*^−/−^ MEFs on retroviral transduction of *TOP1MT* (Figure 1A) but not on transduction with virus particles containing the empty vector (Supplementary Figure S4), which confirms that the increases were caused by TOP1MT deficiency.

In the mammalian nucleus, TOP1 is an essential cofactor of transcription (17,19,26–28). Therefore, the aforementioned observation is counter-intuitive and raises two questions: Which other topoisomerase supports mtDNA transcription in *TOP1MT*^−/−^ MEFs, and why are mtDNA transcripts increased in the absence of TOP1MT?

To address the first question, we measured the abundance of mRNA coding for the other two topoisomerases present in mammalian mitochondria (3,4). We found that TOP2B expression was significantly increased, whereas TOP3A was not significantly altered in *TOP1MT*^−/−^ MEFs as compared with wild-type MEFs (Figure 1C). Given that these mRNAs code for nuclear and mitochondrial enzyme variants (3,4), the observed changes could affect mitochondrial and/or nuclear functions. To differentiate between these possibilities, *TOP2B* was deleted in addition to *TOP1MT*. MEFs deficient in both topoisomerases exhibited mtDNA transcript levels similar to wild-type MEFs and significantly lower than in *TOP1MT*^−/−^ MEFs, whereas mito-biogenesis as judged by mRNA expression of NRF1 and GABPA was stimulated to a similar extent in as in *TOP1MT*^−/−^ MEFs (Figure 1D). In MEFs deficient in TOP2B alone, mtDNA-encoded transcript levels, mRNA markers of the nuclear-driven mito-biogenic stress response (Figure 1D) and expression of TOP1MT and TOP3A (Figure 1C) were the same as in wild-type MEFs. This constellation suggests that neither TOP2B nor TOP1MT are essential for maintaining base line levels of mitochondrial transcription. However, in contrast to TOP2B-deficiency, TOP1MT deficiency is accompanied by a mito-biogenic stress response, which confirms our previous finding (7) that TOP1MT plays a unique and essential role in mtDNA homeostasis that is not complemented by TOP2B and/or TOP3A.

Regarding the second question, we have recently demonstrated that TOP1MT deficiency triggers a nuclear stress response, thereby entailing stimulation of mito-biogenesis (7), which possibly encompasses the observed increase in mtDNA transcripts (29). In keeping with this notion, mRNA markers for the activation of nuclear-driven mito-biogenesis (GABPA, TFAM, POLG) were significantly increased in *TOP1MT*^−/−^ MEFs (7). However, here we found that on *TOP1MT* re-complementation, GABPA mRNA levels failed to normalize along with mtDNA transcript levels (Figure 1A), which suggests that the increase in mtDNA transcripts on TOP1MT deficiency is perhaps not exclusively due to a retrograde stress response of nuclear-driven mito-biogenesis.

**Figure 2.**
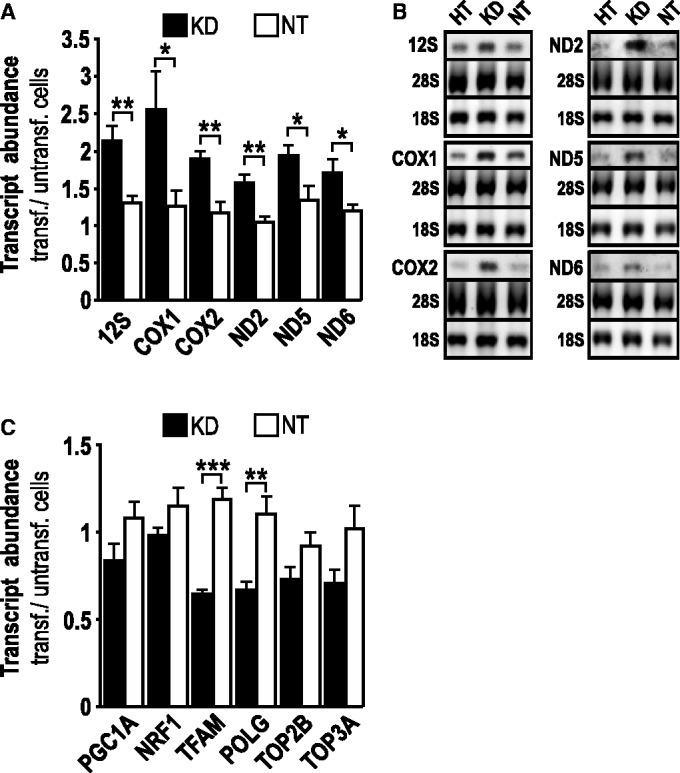
Impact of acute siRNA-mediated depletion of TOP1MT on transcript abundance. (**A**) Quantitative RT-PCR of the indicated mitochondrial transcripts in HT1080 cells transfected with TOP1MT-directed siRNA (KD, black bars) or no target RNA (NT, white bars). (**B**) Northern blot analysis of the indicated mitochondrial transcripts in wild-type cells (HT, left) or cells transfected with TOP1MT-directed siRNA (KD, middle) or no target RNA (NT, right); 28 S and 18 S: nuclear rRNA used as loading control. (**C**) Quantitative RT-PCR of the indicated nuclear transcripts in HT1080 cells transfected with TOP1MT-directed siRNA (KD, black bars) or no target RNA (NT, white bars). Quantitative PCR data are normalized to untransfected cells and given as mean ± SEM, *n *= 5. A representative example for northern blots (*n *= 3) is shown in (B). Full-length northern blots are shown in Supplementary Figure S3.

Next, we tested the alternative hypothesis that a direct negative influence of TOP1MT on mitochondrial transcription and/or transcript stability is released in *TOP1MT*^−/−^ MEFs. This issue was first addressed by studying acute TOP1MT withdrawal in human cells. siRNA-mediated depletion of TOP1MT mRNA and protein by 60 and 90%, respectively, (Supplementary Figure S5) caused a significant increase in the abundance of mitochondrial transcripts across the entire length of heavy strand transcription (12S, COX1, COX2, ND2, ND5) or expressed under the control of the light strand promoter (ND6) (Figure 2A). Electrophoretic mobility of mitochondrial transcripts was not altered (Figure 2B) nor were any aberrant transcripts seen (Supplementary Figure S3), excluding premature or defective termination of transcription. Unlike constitutive *TOP1MT* deficiency, acute siRNA-mediated depletion of TOP1MT was not accompanied by a significant increase in markers of a nuclear mito-biogenic stress response (PGC1A and NRF1) (Figure 2C). Moreover, mRNA levels of TOP2B, TOP3A and nuclear-encoded proteins directly involved in mtDNA transcription (TFAM and POLG) were not significantly altered in response to siRNA-mediated TOP1MT depletion (Figure 2C), as opposed to the increases seen on constitutive deficiency in TOP1MT (Figure 1A) and (7). In summary, these data indicate that acute TOP1MT withdrawal leads to a global increase in mitochondrial transcript abundance without triggering adaptive upregulation of TOP2B or a mito-biogenic stress response in the nucleus. These observations support the hypothesis that the release of a direct negative effect of TOP1MT on mitochondrial transcript stability or transcription rate could be involved rather than a nuclear-driven mito-biogenic stress response to TOP1MT deficiency.

**Figure 3.**
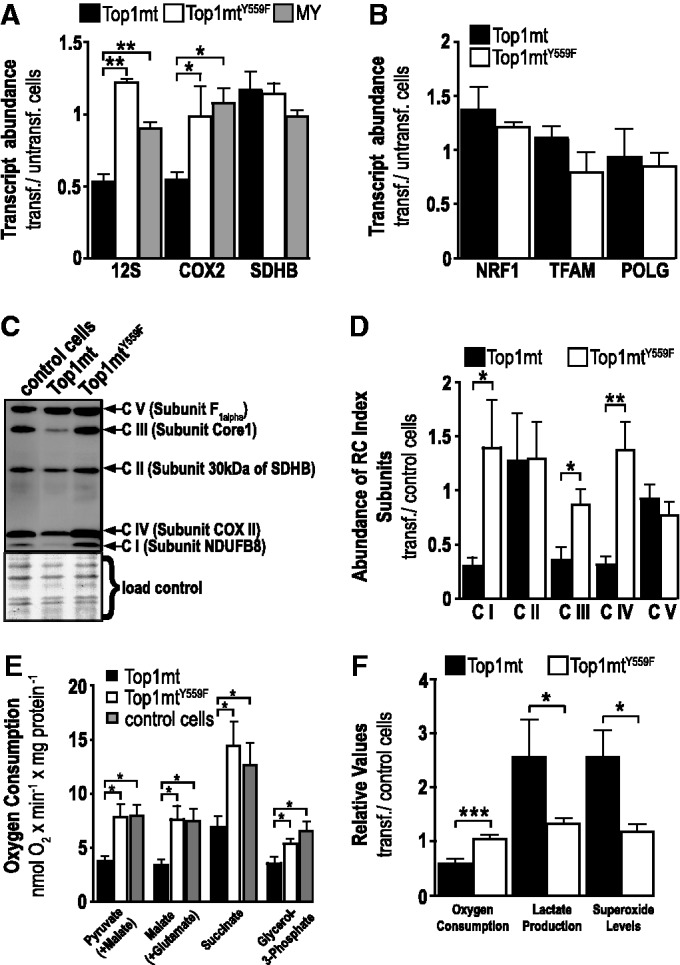
Impact of overexpression of TOP1MT on mtDNA transcripts, respiratory complex proteins and cell respiration. (**A**) Quantitative RT-PCR of the indicated mtDNA transcripts (12 S rRNA, COX1 mRNA) or nuclear encoded transcripts of mitochondrial proteins (SDHB mRNA) in HT1080 cells overexpressing TOP1MT (black) or TOP1MT^Y559F^ (white) or mitochondria targeted YFP serving as vector control (MY, grey); data normalized to control cells and given as mean ± SEM, *n *= 5. (**B**) Quantitative RT-PCR of the indicated mRNA markers of the nuclear mito-biogenesis program in HT1080 cells overexpressing TOP1MT (black) or TOP1MT^Y559F^ (white); data normalized to control cells and given as mean ± SEM, *n *= 5. (**C**) Immunoblot of index subunits of respiratory complexes (CI – CV), representative example of *n *= 5. (**D**) Abundance of index subunits determined by luminometric analysis of immunoblots as shown in (C); data in each lane are normalized to the average signal intensity within the lane and stated as mean ± SEM, *n *= 5. (**E**) Oxygen consumption in control cells (grey) and cells overexpressing TOP1MT (black) or TOP1MT^Y559F^ (white) on addition of the indicated exogenous substrates; mean ± SEM, *n *= 5–7. (**F**) Oxygen consumption (endogenous substrates), lactate production and superoxide levels of cells overexpressing TOP1MT (black) or TOP1MT^Y559F^ (white); data normalized to control cells and given as mean ± SEM, *n *= 3.

To corroborate these findings by a gain-of-function experiment, we stably overexpressed TOP1MT in human cells. A 10-fold increase in TOP1MT mRNA and a corresponding ∼12-fold increase in protein and mtDNA relaxation activity (Supplementary Figure S6A–C) resulted in 20–50% lower levels of mtDNA transcripts (representative examples shown in Figure 3A) and 70% lower levels of index proteins of respiratory complexes I, III and IV, which are dependent on mtDNA-encoded components (Figure 3C and D). mRNA levels of the nuclear-encoded mitochondrial protein SDHB were not affected (Figure 3A). The decrease in abundance of mtDNA transcripts and mtDNA-encoded proteins was clearly due to elevated levels of active TOP1MT because the effect was (i) reverted and even overcompensated on siRNA-mediated depression of TOP1MT-overexpression (Supplementary Figure S6D); (ii) not seen on overexpression of the inactive mutant TOP1MT^Y559F^ at similar levels (Figure 3A, C and D, see Supplementary Figure S6A and B for expression levels); and (iii) not seen on overexpression of mitochondria-targeted YFP (MY) used as vector control (Figure 3A). TOP1MT overexpression did not significantly affect mRNA levels of nuclear encoded proteins associated with mito-biogenesis (NRF1, TFAM, POLG) (Figure 3B) or the relative abundance of index proteins of respiratory complexes II and V, which are devoid of, or partially assembled without, mtDNA-encoded subunits (Figure 3C and D). As a functional consequence of TOP1MT overexpression, cell respiration was decreased by 50%, whereas lactate production and superoxide levels were 2.5-fold increased, and these effects were again not seen on overexpression of the inactive mutant TOP1MT^Y559F^ (Figure 3E and F). In summary, these data support the hypothesis that catalytically active TOP1MT has a negative effect on the abundance of mtDNA transcripts and, as a consequence, the relative abundance of the respiratory complexes dependent on mtDNA-encoded components as well as cell respiration. The data moreover show that suppression of mitochondrial transcripts by TOP1MT is independent of the mito-biogenic nuclear program.

### Selective association of Top1mt with transcriptionally active nucleoids

The proposed direct negative effect of TOP1MT on the abundance of mitochondrial transcripts could be due to a destabilization of the transcripts or an attenuation of transcription rate. The latter mechanism would require a physical interaction with nucleoids undergoing transcription. To test this prediction, YFP bearing a MTS was fused to TOP1MT or TOP1MT^Y559F^ (MY-TOP1MT and MY-TOP1MT^Y559F^), and these constructs (schematized in Supplementary Figure S1) were stably over expressed in HT1080 cells, giving rise to 5-fold increases in TOP1MT-specific mRNA and expression of chimeric proteins of the expected size at levels 8-fold above endogenous TOP1MT. Cells overexpressing MY served as control (Supplementary Figure S7A and B). MY-TOP1MT overexpression induced an increase in mtDNA-relaxation activity corresponding to the overexpression factor at protein level and had a similar impact on the abundance of mtDNA transcripts and respiratory complexes as TOP1MT overexpression. Overexpression of MY or MY-TOP1MT^Y559F^ had no such effect (Supplementary Figure S7C–F). Thus, biological properties of TOP1MT or TOP1MT^Y559F^ were not significantly altered by the YFP attachment.

**Figure 4.**
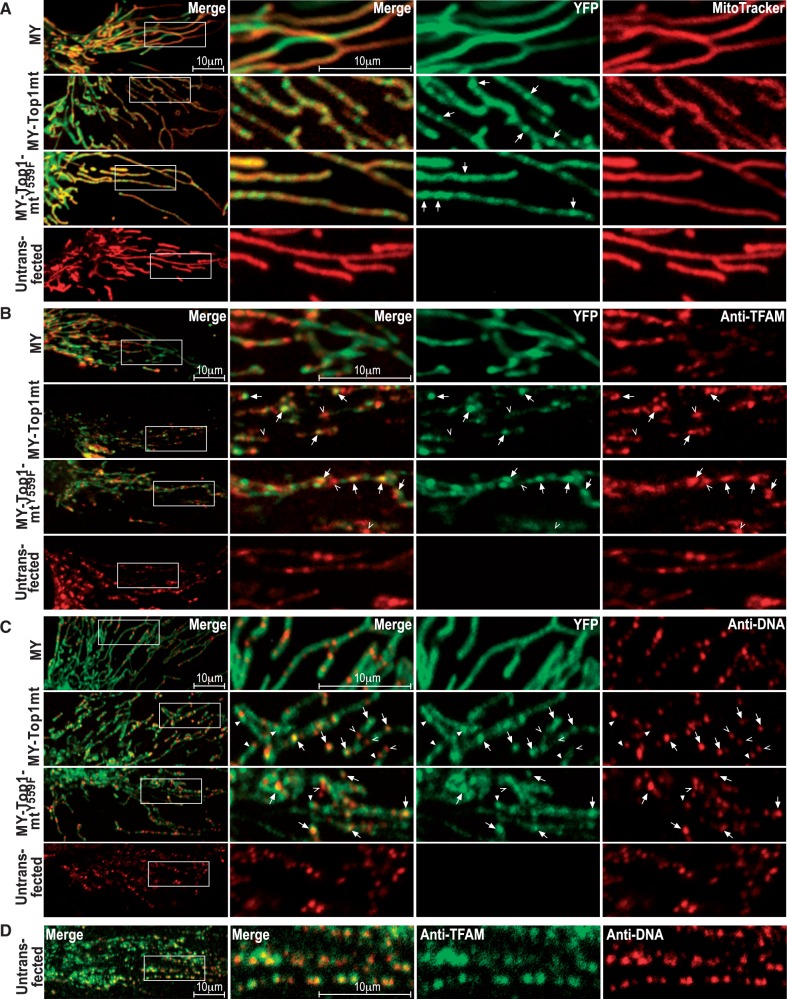
Association of TOP1MT with nucleoids. (**A**) Representative confocal images of live cells expressing YFP-fused constructs as indicated on the left margin. Boxes in leftmost images indicate areas selected for 3-fold enlarged representation in subsequent images to the right. Arrows: examples of MY-TOP1MT and MY-TOP1MT^Y559F^ foci. (**B**, **C**) Similar cells as in (A) fixed and counterstained with TFAM (B) or DNA (C) antibodies. Arrows: foci of TFAM or DNA co-localized with MY-TOP1MT or MY-TOP1MT^Y559F^; open arrowheads: foci of TFAM or DNA not co-localized with MY-TOP1MT or MY-TOP1MT^Y559F^; closed arrowheads in (C): ‘DNA-free’ foci of MY-TOP1MT or MY-TOP1MT^Y559F^. (**D**) Untransfected cells co-stained with antibodies against TFAM (middle right) and DNA (right). Size bars given in the two leftmost images at the top also apply to all subsequent images of the respective section.

Cells overexpressing the fluorescent constructs all exhibited a typical mitochondrial network (30) with branching and interconnectivity similar to control cells excluding major effects on mitochondrial fusion/fission equilibrium (Figure 4A, left). All three fluorescent proteins were strictly intra-mitochondrial but had a different distribution: MY was homogeneously distributed similar to MitoTracker Red, indicating that it was freely diffusible in the mitochondrial matrix (Figure 4A). In contrast, MY-TOP1MT accumulated at intra-mitochondrial foci (Figure4A, arrows). The inactive mutant MY-TOP1MT^Y559F^ (Figure 4A, arrows) formed fewer and less intense foci (Supplementary Figure S8A), suggesting that focal accumulation of TOP1MT did not require, but was enhanced by catalytic activity. The true size of the foci was determined by super-resolution microscopy (Supplementary Figure S8B). It was similar for MY-TOP1MT and MY-TOP1MT^Y559F^ (72 ± 28 and 66 ± 33 nm, respectively) and conforms to the size of mammalian nucleoids (12).

To confirm that these structures were nucleoids, we counterstained cells with antibodies against TFAM and DNA. TFAM showed a typical focal pattern within mitochondria (31) that was similar in overexpressing and control cells indicating that overexpression of MY-TOP1MT or MY-TOP1MT^Y559F^ had no major effect on general nucleoid composition (Figure 4B). All MY-TOP1MT foci were co-localized with TFAM foci (Figure 4B, arrows), whereas about one-third of the more numerous TFAM foci were not co-localized with MY-TOP1MT foci (Figure 4B, open arrowheads). Analogous results were obtained with MY-TOP1MT^Y559F^ (Figure 4B). Consistent with published findings (12), TFAM-signals completely overlapped with DNA-signals (Figure 4D). Only about two-thirds of the DNA-foci were co-localized with MY-TOP1MT foci (Figure 4C, arrows), whereas some DNA foci were negative for MY-TOP1MT (Figure 4C, open arrowheads). A minor fraction of MY-TOP1MT appeared devoid of DNA (Figure 4C, closed arrowheads). EtBr-induced mtDNA depletion resulted in complete disappearance of all MY-TOP1MT foci (Supplementary Figure S9), suggesting that their formation is DNA-dependent, while undetectable small amounts of DNA might be present at some TOP1MT foci. In conclusion, focal accumulation of TOP1MT reflects association with nucleoids, but TOP1MT seems to associate with only two-thirds of the nucleoids delineated by TFAM and DNA.

**Figure 5.**
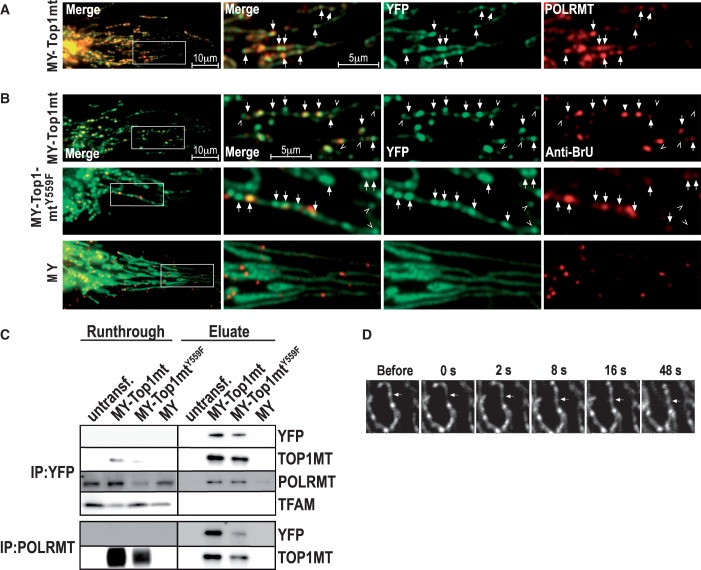
Selective interaction of TOP1MT with transcriptionally active nucleoids and POLRMT. (**A**, **B**) Cells expressing MY-TOP1MT stained with POLRMT antibodies (A) or expressing MY-TOP1MT, MY-TOP1MT^Y559F^ or MY stained with BrU antibodies following incubation with 2.5 mM BrU (B); corresponding images of YFP-specific fluorescence (middle right), antibody-specific fluorescence (right) and merged signals (middle left and left) are representative of the whole-cell population. Boxes in the overviews (left) indicate areas selected for 3-fold enlarged representation; arrows: MY-TOP1MT foci co-localizing with POLRMT or BrU; open arrowheads: MY-TOP1MT foci not co-localized with BrU. (**C**) Co-immunoprecipitation of TOP1MT or TOP1MT^Y559F^ with POLRMT. Lysates of untransfected cells or cells expressing MY, MY-TOP1MT or MY-TOP1MT^Y559F^ were subjected to YFP- (top) or POLRMT-directed (bottom) immunoprecipitation. Equivalent amounts of run through (left) and immune-precipitate (right) were probed with antibodies against YFP, TOP1MT, POLRMT or TFAM as indicated on the right margin. The result is representative of three identical experiments with similar result. (**D**) Exchange rate of MY-TOP1MT molecules at nucleoids analysed by fluorescence recovery after photo bleaching. Images of a selected mitochondrial segment acquired before and at the indicated time points after bleaching a selected MY-TOP1MT focus (arrows).

To test whether the subset of nucleoids positive for MY-TOP1MT were undergoing transcription, we counterstained cells overexpressing MY-TOP1MT with antibodies against POLRMT, which decorated typical mitochondrial foci (10,32) that co-localized with MY-TOP1MT foci (Figure 5A, arrows). To confirm that such foci were active transcription sites, we cultured the cells with BrU and detected newly synthesized RNA with BrU-antibodies. All foci positive for BrU-RNA were also positive for MY-TOP1MT (Figure 5B, top, arrows). Conversely, only two-thirds of MY-TOP1MT foci were positive for BrU (Figure 5B, top, open arrowheads), suggesting that TOP1MT selectively associated with nucleoids containing POLRMT, but not all these nucleoids were equally active in terms of RNA synthesis. Thus, TOP1MT seems to associate with nucleoids containing POLRMT even when transcription-related DNA- or RNA-structures are absent, which is reminiscent of the constitutive association of TOP1 with RNA polymerase I in the nucleus (15). Consistent with this notion, TOP1MT was co-immunoprecipitated with POLRMT but not TFAM (Figure 5C), indicating a selective and specific physical association with the transcription machinery. POLRMT co-immunoprecipitation was also observed with TOP1MT^Y559F^ and therefore independent of TOP1MT activity. In keeping with this notion, MY-TOP1MT^Y559F^ exhibited a focal co-localization with BrU-RNA similar to MY-TOP1MT (Figure 5B, middle), whereas MY did not (Figure 5B, bottom). BrU-RNA foci co-localized with MY-TOP1MT^Y559F^ appeared to be slightly more intense than those co-localized with MY-TOP1MT, which conforms to the notion that only the active enzyme has a negative effect on the abundance of mtDNA transcripts. This could not be confirmed in cells expressing MY because there the BrU-signal was to some extent quenched by the high level of YFP-fluorescence in the surrounding matrix (Figure 5B, bottom).

### Rapid exchange of Top1mt at nucleoids

There is little exchange of mtDNA between nucleoids (9). Therefore, selective association of TOP1MT with nucleoids containing POLRMT suggested by the above data would require constant repositioning of the enzyme inside mitochondria, as individual nucleoids alter their transcriptional state. To test this prediction, MY-TOP1MT fluorescence was bleached at a selected focus and fluorescence recovery at that focus was monitored (Figure 5D). MY-TOP1MT fluorescence at the bleached focus (arrow) mostly recovered within a minute. As bleaching is irreversible, the observed recovery is due to MY- TOP1MT molecules moving in from the unbleached neighbourhood. These results indicate that TOP1MT has a sufficient mobility and exchange rate to maintain a selective association with a constantly changing subset of nucleoids. We previously demonstrated a similar mechanism of rapid scanning and transient focal accumulation for the association of TOP1 with nuclear rDNA transcription sites (16). However, the N-terminal domain directing TOP1 to such sites (33) is absent in TOP1MT (5).

## Concluding remarks

The following conclusions can be drawn from the data presented in this study: (i) Neither TOP2B nor TOP1MT are essential for maintaining base line levels of mitochondrial transcription. However, TOP1MT has an additional function in mtDNA homeostasis (7) that is not complemented by the other mitochondrial topoisomerases. (ii) TOP1MT has a direct dominant negative effect on mitochondrial transcript abundance, which depends on the enzymatic activity and is matched by a physical association with transcriptional active nucleoids and POLRMT. (iii) Together, these conclusions suggest that the negative impact on mitochondrial transcript abundance could be a major biological function of TOP1MT. (iv) TOP1MT decreases mtDNA transcript abundance, irrespective of the distance between transcript and promoter or the requirement of POLRMT to translocate through the D-loop region during transcription. (v) The negative impact of TOP1MT on mitochondrial transcript abundance influences the balance between nuclear and mtDNA-encoded respiratory subunits in a manner relevant for cell respiration.

In summary, TOP1MT is suggested as a global negative regulator either of the mitochondrial transcription rate or the stability of mtDNA transcripts. We could not distinguish yet between these two possible mechanisms because we measured transcript abundance and not transcription rate. The latter parameter can in principle be assessed *in vitro* (14,34), but in the present study, that approach was compromised by the fact that nuclear TOP1 is an obligatory contaminant of TOP1MT preparations and a powerful inhibitor of mitochondrial transcription (8). Therefore, we could not have possibly distinguished whether a negative effect of purified TOP1MT on mtDNA transcription *in vitro* is due to TOP1MT itself or contaminating traces of TOP1.

The inhibitory function of TOP1MT on mtDNA transcription demonstrated here is in striking contrast to the stimulatory role of TOP1 on nuclear transcription. TOP1 activity is known to be essential for the promotion of nuclear mRNA (17,35–37) and rRNA transcription (2,18,19), while a dampening effect of TOP1 on basal transcription (19,26) and an auxiliary role in the assembly of RNA polymerase II transcription complexes (27) is independent of catalytic activity. These functional differences suggest that the conserved evolutionary splitting of the *TOP1* gene into nuclear and mitochondrial paralogs (5,6) is mirrored by a divergent specialization of transcription-associated functions. The argument is supported by our previous observation, that aberrant targeting of TOP1 to mitochondria completely disrupts mtDNA transcription (8). On the other hand, it has been suggested that a nuclear isoform of POLRMT may be able to transcribe certain nuclear genes (38). As TOP1MT is working in concert with POLRMT in the mitochondria as shown here, it could also be involved in such nuclear tasks of POLRMT. However, we have previously observed that TOP1MT is incapable of interacting with nuclear chromatin (8), which seems to argue against such a role.

The core machinery required for mammalian mtDNA transcription initiation *in vitro* is composed of TFAM, POLRMT and the rRNA methyltransferase-related transcription factor B2 (14,39). This complex has additional transient constituents that control various aspects of the transcription process (40,41). Based on our data, it seems conceivable that TOP1MT is another such transient component and exerts a depressive influence on mitochondrial transcripts via direct interference with transcription initiation, similar to the function of TOP1 in the assembly of the TFIID-TFIIA complex during activation (27) or repression (26) of nuclear mRNA transcription. However, these regulatory functions of TOP1 in nuclear transcription initiation are independent of catalytic activity, whereas the negative effect of TOP1MT on mitochondrial transcripts is not. Therefore, it seems more likely that the latter effect is related to the removal of supercoils from mtDNA. In nuclear transcription, TOP1 removes positive supercoils generated ahead of moving transcription complexes (2) and releases negative supercoils behind the transcription machinery, which would otherwise form R-loops with the nascent transcript (35–37). These activities serve to promote nuclear transcription elongation. It is not easy to envision how TOP1MT could have the opposite effect on mitochondrial transcription by removing positive or negative supercoils from mtDNA. One conceivable scenario would be that a certain degree of basal mtDNA supercoiling is a prerequisite for mitochondrial transcription initiation. TFAM is known to compact mtDNA in a process bending the DNA backbone (42). The resulting mtDNA conformation is assumed to harbor multiple U-turns, which are required for the proper assembly of the transcription complex on the DNA (43). It is probable that the compaction process introduces supercoils into mtDNA, and that the proposed U-turn structure becomes less stable when mtDNA supercoiling is reduced. On these premises, TOP1MT activity could negatively regulate mitochondrial transcription by releasing mtDNA supercoils and thereby loosening or abolishing TFAM-induced U-turn conformations required for transcription initiation.

However, irrespective of the precise mechanism, we clearly demonstrate that experimental manipulations of TOP1MT expression lead to inverse changes in mtDNA transcripts levels that have a measurable impact on cell respiration. Physiological upregulation of TOP1MT has been observed in cellular stress responses encompassing the induction of mitochondrial biogenesis (29) and in cancer cells, where TOP1MT is induced by the proto-oncogene MYC (44). Thus, a dominant negative effect of TOP1MT on mitochondrial transcript abundance and cell respiration could plausibly be involved in stress adaptation of respiratory capacity and in the downregulation of cell respiration in cancer cells (the Butenant effect).

## SUPPLEMENTARY DATA

Supplementary Data are available at NAR Online.

## FUNDING

Deutsche Forschungsgemeinschaft [SFB 728 and GRK 1033 to F.B., DFG Wi 889/6-2 to R.J.W.] and the National Institutes of Health, National Cancer Institute, Center for Cancer Research [Intramural Research Program to Y.P.]. Funding for open access charge: Research budget of the Institute of Clin. Chemistry and Lab. Diagnostics, Univ. Dusseldorf, of which the corresponding author is the director.

*Conflict of interest statement*. None declared.

## Supplementary Material

Supplementary Data
