# Supplementary material for: Negative regulation of mitochondrial transcription by mitochondrial topoisomerase I
Source: Nucleic Acids Res. 2013 Aug 27;41(21):9848–57. doi: 10.1093/nar/gkt768 (PMC3834834; doi:10.1093/nar/gkt768)
Supplement: Supplementary Data [file supp_41_21_9848__index.html]

Negative regulation of mitochondrial transcription by mitochondrial topoisomerase I — Supplementary Data 

# Negative regulation of mitochondrial transcription by mitochondrial topoisomerase I

## Supplementary Data

files

**Files in this Data Supplement:**

- Supplementary Data - pdf file
