# Supplementary material for: Negative regulation of mitochondrial transcription by mitochondrial topoisomerase I
Source: Nucleic Acids Res. 2013 Aug 27;41(21):9848–57. doi: 10.1093/nar/gkt768 (PMC3834834; doi:10.1093/nar/gkt768)
Supplement: Supplementary Data [file supp_gkt768_nar-02980-h-2012-File007.pdf]

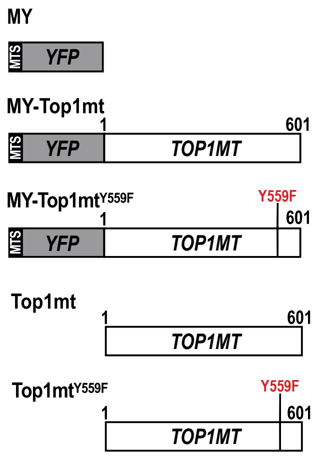

Suppl. Fig. 1. Constructs for constitutive overexpression of TOP1MT. “MTS” indicates the position of a mitochondrial targeting sequence derived from the COX1 gene.

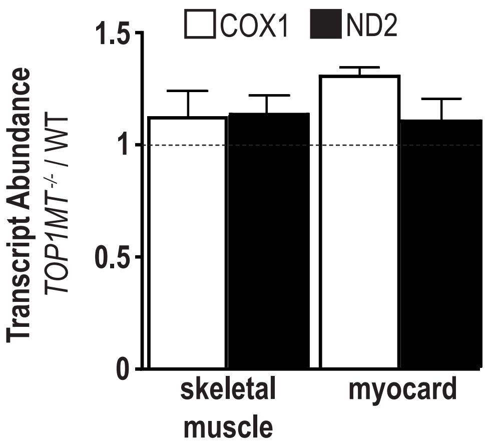

Suppl. Fig.2. mtDNA transcript abundance in skeletal muscle and myocard of *TOP1MT*$^{-/-}$ mice. Quantitative RT-PCR of COX1 mRNA (white bars) and ND2 mRNA (black bars) in the indicated tissues of *TOP1MT*$^{-/-}$ mice; data are normalized to corresponding values obtained in *TOP1MT*$^{+/+}$ littermates and stated as mean ± SEM, n=5.

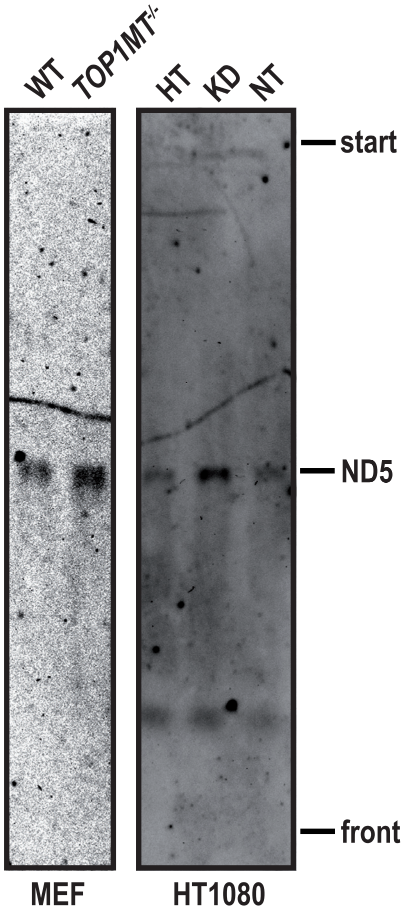

Suppl. Fig. 3. Full length representation of Northern blot analysis. Images correspond to the data shown for ND5 in Fig. 1 B (left panel) and Fig. 2 B (right panel).

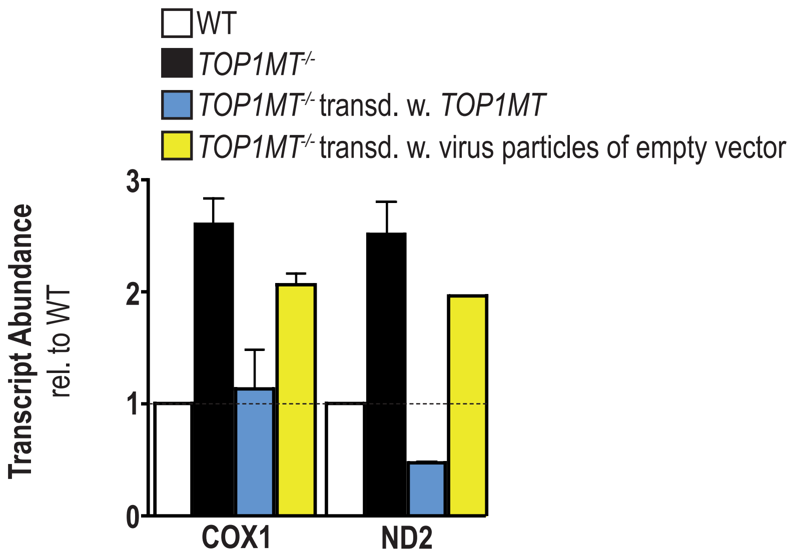

Suppl. Fig. 4. Vector control for retroviral recomplementation of *TOP1MT*$^{-/-}$ MEFs. Quantitative RT-PCR of COX1 mRNA (left) and ND2 mRNA (right) in wild type MEFs (white bars) or *TOP1MT*$^{-/-}$ MEFs (black bars) optionally transduced with *TOP1MT* (blue bars) or virus particles of empty vector (yellow bars); data normalized to values obtained in wildtype MEFs from *TOP1MT*$^{+/+}$littermates (dashed line) are stated as mean ± SEM, n=3.

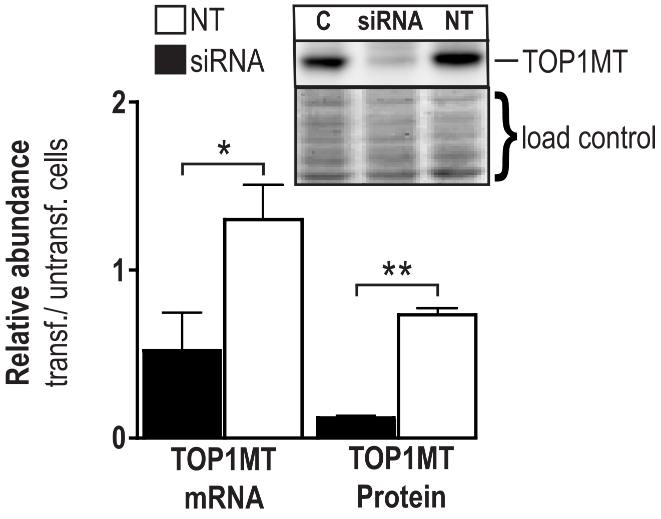

Suppl. Fig. 5. siRNA-mediated depletion of TOP1MT mRNA and protein. Quantitative RT-PCR of TOP1MT-specific mRNA (left) and protein (right) in HT1080 cells transfected with siRNA targeting TOP1MT (black bars) or non-targeting control siRNA (white bars); data normalized to values obtained in untransfected cells are stated as mean ± SEM, n=5; Insert: Representative example of TOP1MT-directed immunoblot (top) and corresponding load control (bottom) in control cells (C) or cells transfected with siRNA targeting TOP1MT (siRNA) or non-targeting control siRNA (NT).

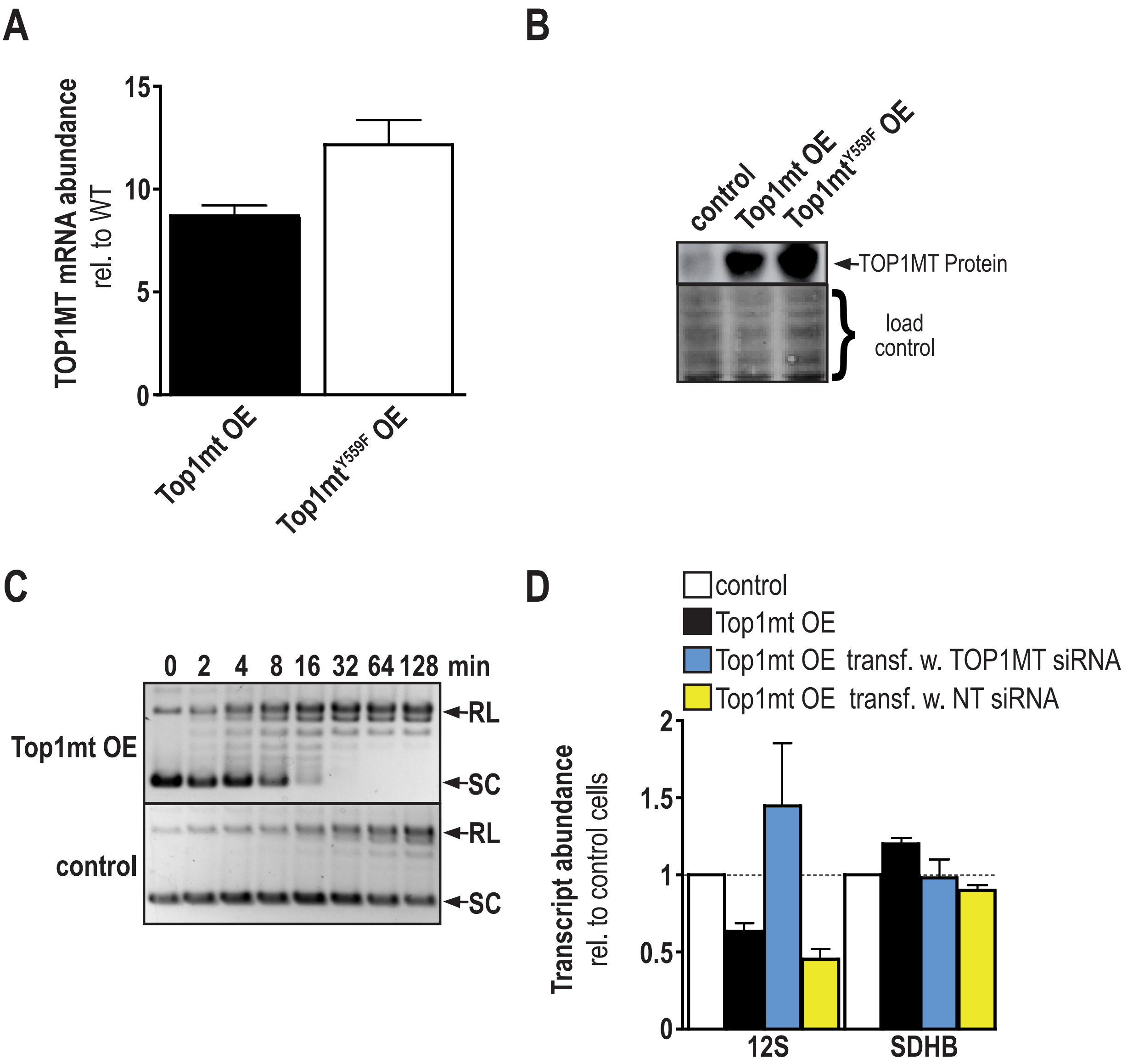

Suppl. Fig. 6. Characteristics of HT1080 cells overexpressing TOP1MT or TOP1MT^{Y559F}. A.) Quantitative RT-PCR of TOP1MT-specific mRNA in HT1080 cells constitutively overexpressing human TOP1MT (black) or TOP1MT^{Y559F} (white); data normalized to values obtained in control cells are stated as mean ± SEM, n=5. B.) Representative example of a TOP1MT-directed immunoblot of HT1080 control cells or HT1080 cells constitutively overexpressing human TOP1MT or TOP1MT^{Y559F}. C.) Mitochondrial DNA relaxation activity in HT1080 cells constitutively overexpressing TOP1MT (top) or not (bottom). Standardized mitoplast extracts were incubated with pUC18 plasmid DNA for the time intervals indicated. DNA supercoiling was determined by gel electrophoresis and EtBr-staining. The position of relaxed (RL) and supercoiled (SC) DNA bands is indicated on the right margin. Representative result of three analyses with similar outcome. D.) Quantitative RT-PCR of 12S rRNA (left) and SDHB mRNA (right) in control HT1080 cells (white bars), cells overexpressing TOP1MT (black bars) and optionally subjected to reversal of overexpression by transfection with siRNA targeting TOP1MT (blue bars) or transfection with non-targeting control siRNA (yellow bars).

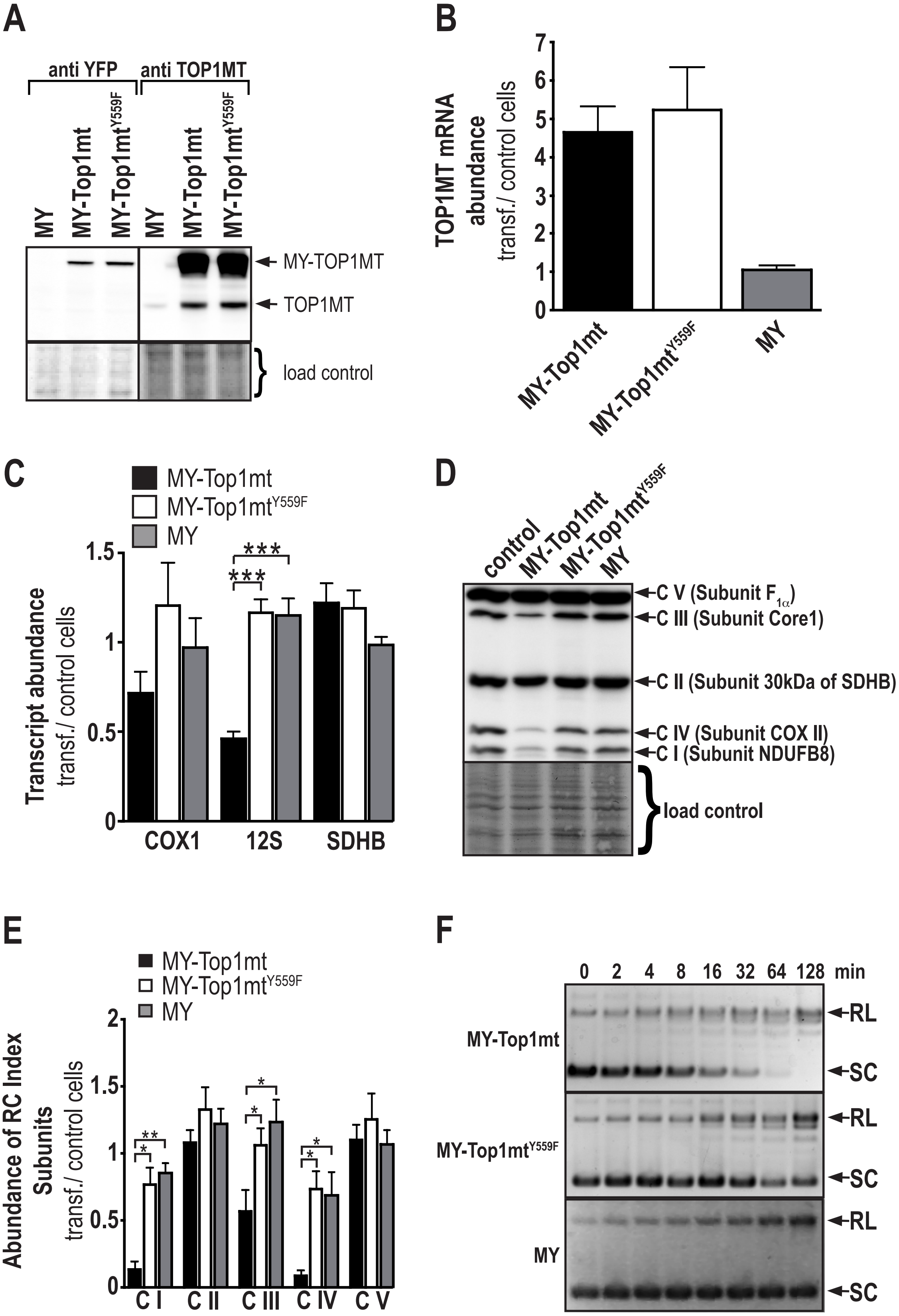
A
anti YFP
anti TOP1MT
MY
MY-Top1mt
MY-Top1mtY559F
MY-TOP1MT
TOP1MT
load control
B
TOP1MT mRNA abundance
transf./ control cells
0
1
2
3
4
5
6
7
MY-Top1mt
MY-Top1mtY559F
MY
C
MY-Top1mt
MY-Top1mtY559F
MY
Transcript abundance
transf./ control cells
0
0.5
1
1.5
***
***
COX1
12S
SDHB
D
control
MY-Top1mt
MY-Top1mtY559F
MY
C V (Subunit F1α)
C III (Subunit Core1)
C II (Subunit 30kDa of SDHB)
C IV (Subunit COX II)
C I (Subunit NDUFB8)
load control
E
MY-Top1mt
MY-Top1mtY559F
MY
Abundance of RC Index Subunits
transf./ control cells
0
0.5
1
1.5
2
**
*
*
*
*
*
C I
C II
C III
C IV
C V
F
0
2
4
8
16
32
64
128
min
MY-Top1mt
MY-Top1mtY559F
MY
RL
SC

Suppl. Fig. 7. Characteristics of cells overexpressing YFP-fused TOP1MT constructs. A.) TOP1MT-directed (top, right) and YFP-directed immunoblot (top, left) and corresponding load controls (bottom) of HT1080 cells overexpressing the indicated YFP-fused constructs; arrows: expected migration distances of TOP1MT and MY-TOP1MT. B.) Quantitative RT-PCR of TOP1MT-specific mRNA in cells overexpressing the indicated YFP-fused constructs; data normalized to control cells are stated as mean ± SEM of four individual cell clones. C.) Quantitative RT-PCR of mtDNA-encoded (COX1 mRNA, 12S rRNA) or nuclear encoded (SDHB mRNA) transcripts in cells overexpressing MY-Top1mt (black), MY-Top1mt^{Y559F} (white) or MY (grey); data normalized to control cells are stated as mean ± SEM, n=5. D.) Representative immunoblot (top) of index subunits of respiratory complexes (CI – CV, as indicated on the right margin) and corresponding load control (bottom) in control cells or cells overexpressing the indicated YFP-fused constructs. E.) Abundance of index subunits determined in cells overexpressing MY-TOP1MT (black) or MY-TOP1MT^{Y559F} (grey) or MY (white) by luminometric analysis of immunoblots such as shown in D; data in each lane are normalized to the average signal intensity within the lane and stated as mean ± SEM, n=4. F.) DNA relaxation activity in mitoplast extracts of cells constitutively overexpressing MY-TOP1MT (top), MY-TOP1MT^{Y559F} (middle) or MY (bottom) determined as described in legend to Suppl. Fig. 6C, representative example of four independent experiments with similar outcome.

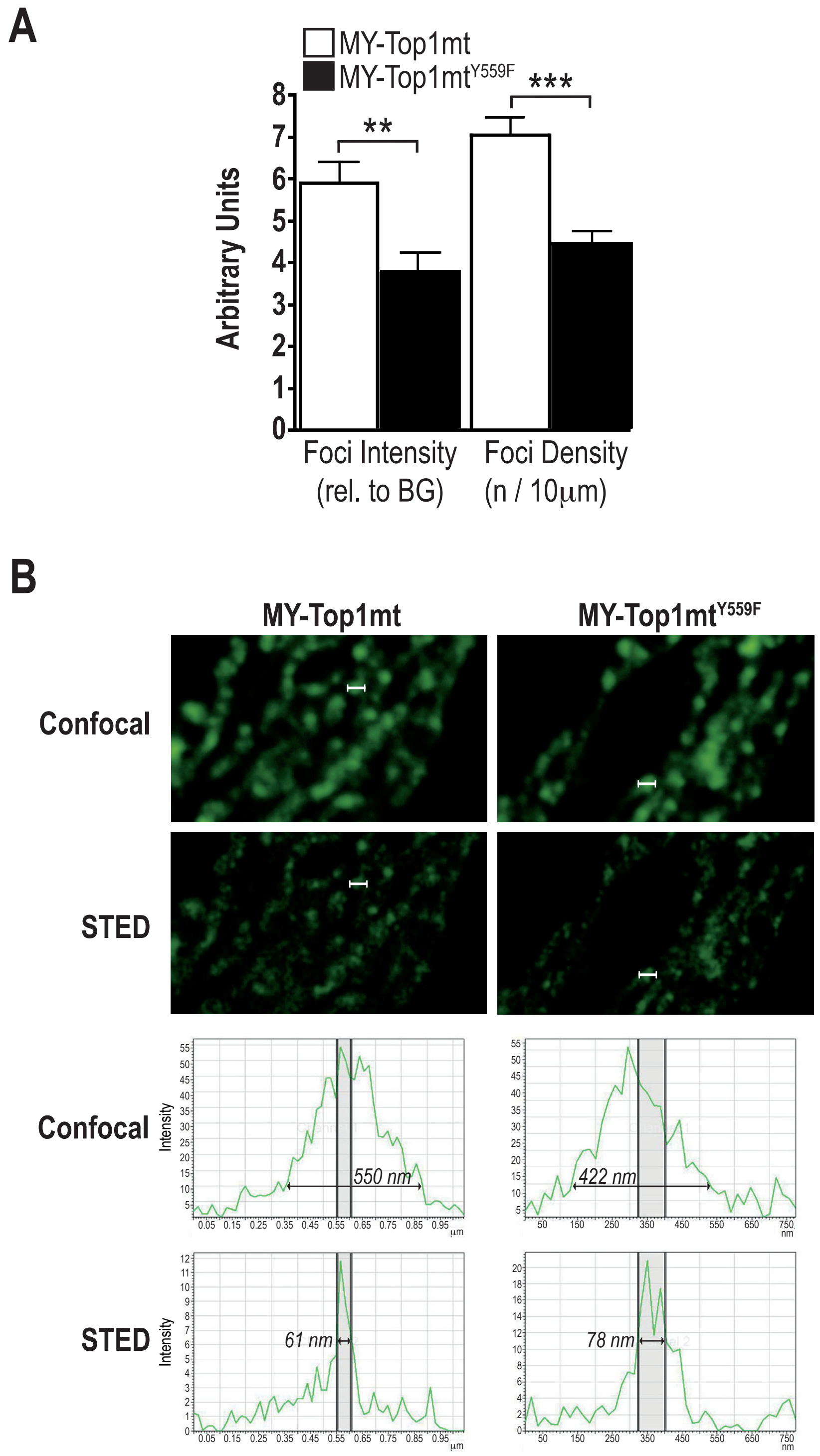

Suppl. Fig. 8. Intensity, frequency and true size of intra-mitochondrial TOP1MT foci. A.) Fold increases in fluorescence intensity in a focus relative to the adjacent background (leftmost two columns) and the number of foci along a 10 µm stretch of mitochondrial tubule (rightmost two columns) determined in confocal images of cells overexpressing MY-TOP1MT (white columns) or MY-TOP1MT^{Y559F} (black columns). At least 20 foci or two 10 µm stretches of mitochondrial tubule, respectively, were analysed in each cell. Mean values ± SD from 10 individual cells are stated. B.) Images of YFP-fluorescence obtained by confocal microscopy (top) and STED-microscopy (bottom) of representative mitochondrial areas in cells overexpressing MY-TOP1MT (left) and MY-TOP1MT^{Y559F} (right). The screen-prints from the microscope software shown at the bottom demonstrate the fluorescence intensity distribution across a single focus marked by the tracks in the fluorescent images. Estimates of the apparent size of the foci in confocal (top) and STED (bottom) images are indicated by yellow bars. The true focus size derived from the analysis of at least 10 foci in various areas of the cell was 72 ± 28 and 66 ± 33 nm for foci formed by MY-TOP1MT and MY-TOP1MT^{Y559F}, respectively.

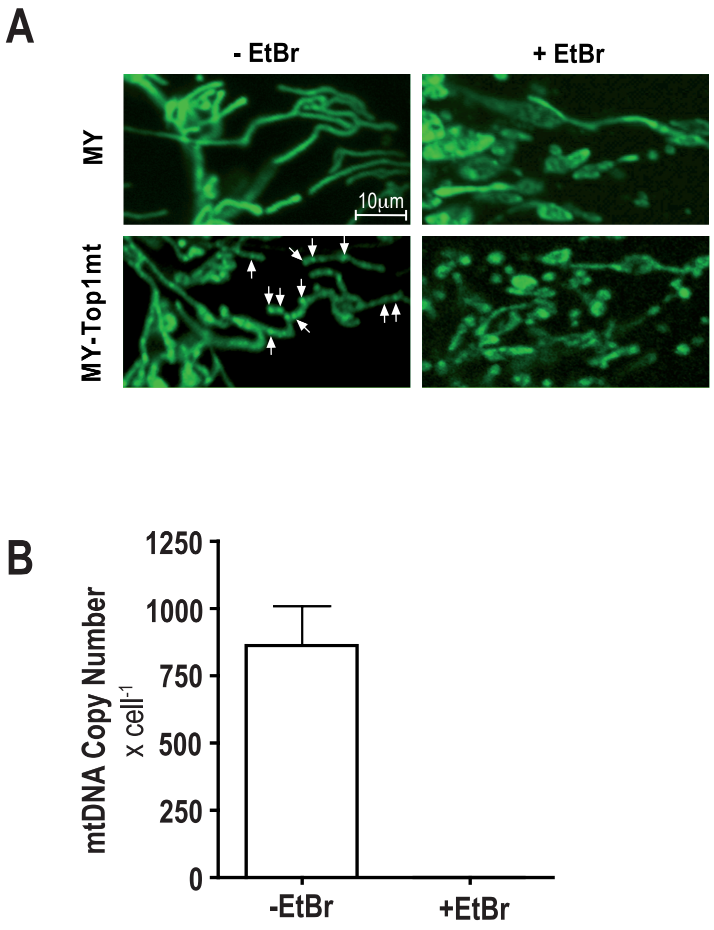

Suppl. Fig. 9. mtDNA-dependence of focal intra-mitochondrial MY-TOP1MT accumulation. A.) Confocal images of cell clones stably overexpressing MY (top) or MY-TOP1MT (bottom) grown for 2 weeks in the absence (left) or presence (right) of 50 ng/ml EtBr and 50 µg/ml uridine. B.) mtDNA content of the cells determined by quantitative RT PCR as described in ref. (7).
